# Supplementary figures and images for: Gravidity influences distinct transcriptional profiles of maternal and fetal placental macrophages at term
Source: Front Immunol. 2024 Jun 26;15:1384361. doi: 10.3389/fimmu.2024.1384361 (PMC11237841; doi:10.3389/fimmu.2024.1384361)

## Supplementary Figure 1.

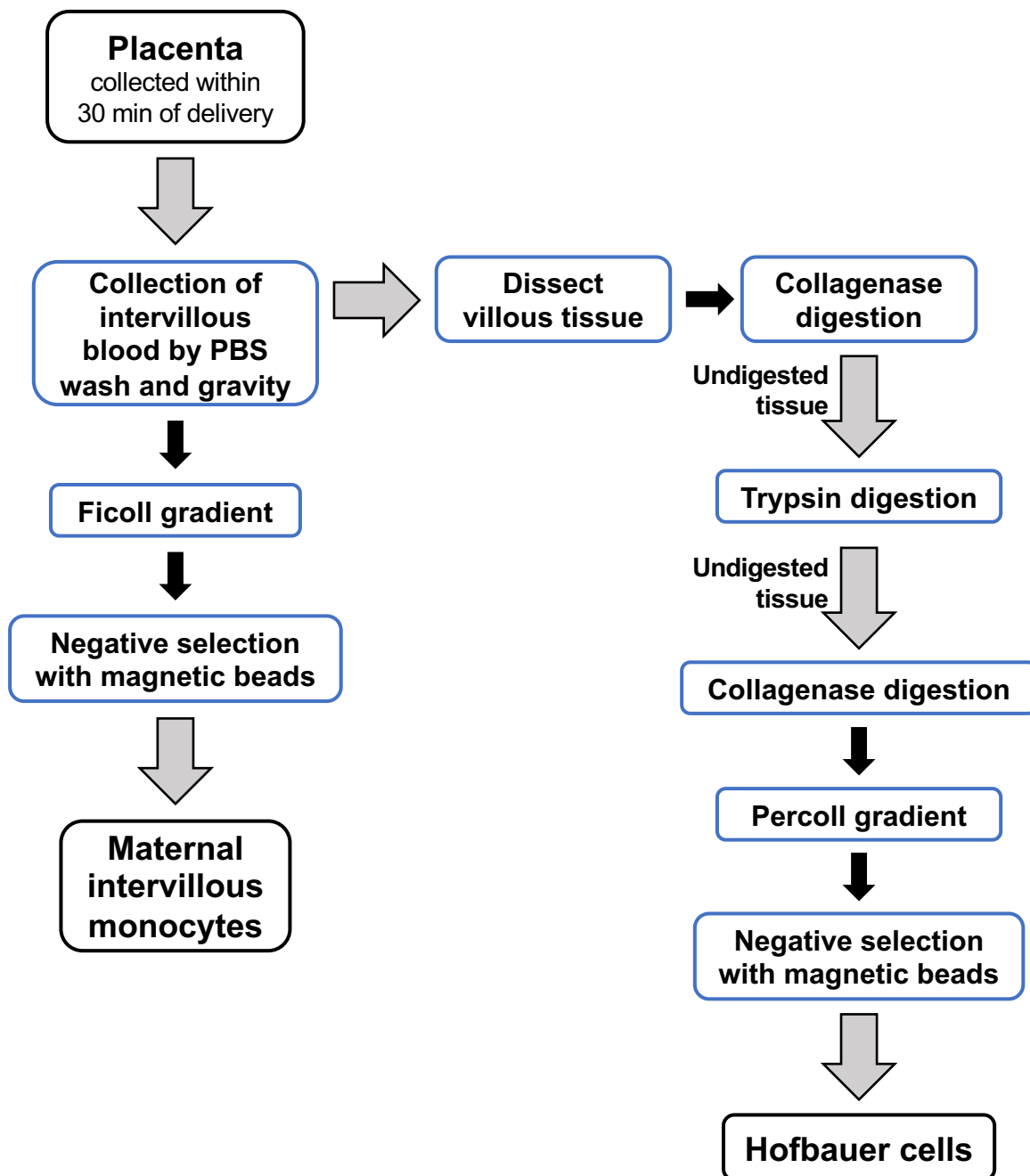

Supplement: Supplementary file 1 [file DataSheet_1.pdf]
