# Supplementary material for: Gravidity influences distinct transcriptional profiles of maternal and fetal placental macrophages at term
Source: Front Immunol. 2024 Jun 26;15:1384361. doi: 10.3389/fimmu.2024.1384361 (PMC11237841; doi:10.3389/fimmu.2024.1384361)

Supplementary Figure 2. Hierarchical clustering of Hofbauer cell markers defined by Vento-Tormo et. al.
(**A**) Out of 30 markers identified in the single cell RNA-seq study of Vento-Tormo et.al, 19 were found to be significant according to our analysis (adj-p<0.01). Fold changes for each sample were calculated according to average MIM value. (**B**) Using the significant 19 markers, a new fold change between HBCs-MIMs was calculated for each sample pair.

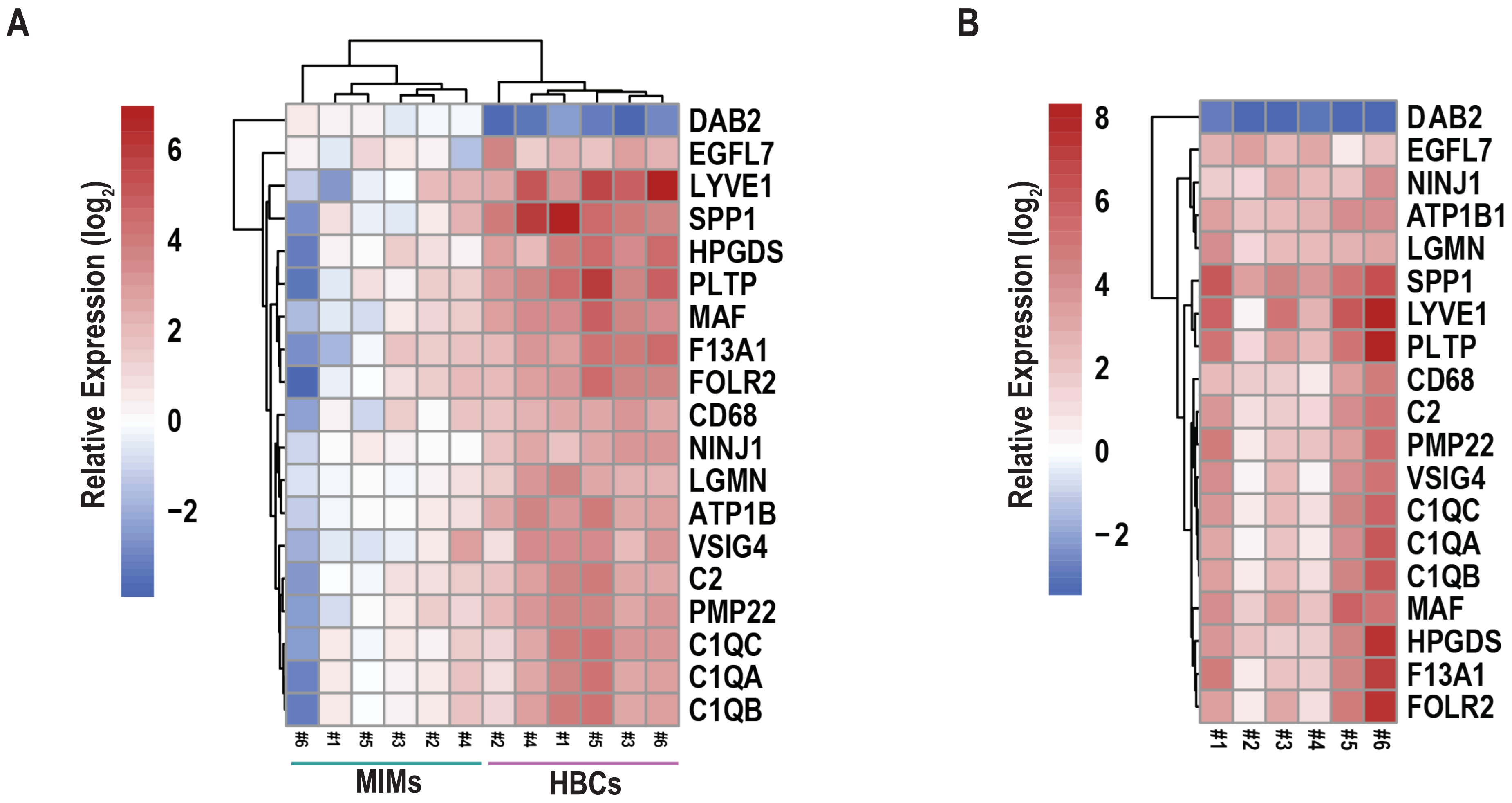

Supplement: Supplementary file 2 [file DataSheet_2.pdf]
