# Supplementary material for: Gravidity influences distinct transcriptional profiles of maternal and fetal placental macrophages at term
Source: Front Immunol. 2024 Jun 26;15:1384361. doi: 10.3389/fimmu.2024.1384361 (PMC11237841; doi:10.3389/fimmu.2024.1384361)
Supplement: Supplementary file 3 [file Table_1.pdf]

Supplementary Table 1. Patient characteristics and birth outcomes and experimental usage.													
Patient ID	Maternal age (years)	Gestational age (weeks)	Gravidity	Mode of Delivery	Birth weight (gram)	Low birth weight (<2500 g)	Small for gestational age	Infant Sex	MIM count (cells/ml)	HBC count (cells/gr placental tissue)	RNA-seq experiment	qRT-PCR experiment	
												MIMs	HBCs
#1	22	41.0	Multigravida	Vaginal	3450	No	No	Male	N/A	21,714	Yes	Yes	No
#2	18	39.9	Primigravida	Vaginal	2950	No	No	Female	4,000,000	16,000	Yes	Yes	No
#3	27	40.6	Multigravida	Vaginal	3580	No	No	Female	6,090,000	9,310	Yes	Yes	No
#4	18	41.3	Primigravida	Cesarean	3140	No	No	Male	60,000,000	6,214	Yes	Yes	Yes
#5	26	40.6	Multigravida	Vaginal	2830	No	No	Male	7,700,000	42,830	Yes	Yes	Yes
#6	18	37.1	Primigravida	Vaginal	2670	No	No	Male	36,375,000	47,000	Yes	Yes	No
#7	20	40.6	Primigravida	Cesarean	2800	No	No	Male	6,000,000	7,727	No	No	Yes
#8	29	38.0	Multigravida	Vaginal	2230	Yes	Yes	Male	10,400,000	37,037	No	Yes	Yes
#9	22	41.1	Multigravida	Vaginal	2620	No	Yes	Female	87,000,000	242,424	No	Yes	Yes
#10	29	39.0	Multigravida	Vaginal	2520	No	Yes	Male	17,000,000	8,824	No	Yes	Yes
#11	22	39.4	Multigravida	Vaginal	3400	No	No	Male	16,000,000	17,217	No	Yes	Yes
#12	25	39.0	Multigravida	Vaginal	2780	No	No	Male	N/A	6,494	No	Yes	No
#13	22	40.3	Multigravida	Vaginal	2850	No	No	Female	12,000,000	18,783	No	Yes	Yes
#14	19	41.1	Multigravida	Cesarean	2850	No	Yes	Female	6,000,000	N/A	No	Yes	Yes
#15	26	40.9	Multigravida	Vaginal	3090	No	No	Male	7,500,000	N/A	No	Yes	Yes
#16	20	37.7	Multigravida	Vaginal	2880	No	No	Female	N/A	12,000	No	Yes	Yes
#17	28	39.0	Multigravida	Vaginal	3029	No	No	Male	117,000,000	4,000	No	Yes	No
#18	31	39.0	Multigravida	Vaginal	3420	No	No	Male	6,250,000	N/A	No	Yes	Yes
#19	27	43.0	Multigravida	Vaginal	3120	No	No	Male	15,000,000	17,097	No	Yes	Yes
#20	25	39.0	Multigravida	Vaginal	3150	No	No	Male	4,812,500	21,203	No	Yes	Yes
#21	22	39.4	Multigravida	Vaginal	2900	No	No	Male	6,500,000	7,734	No	No	Yes
