# Supplementary material for: Gravidity influences distinct transcriptional profiles of maternal and fetal placental macrophages at term
Source: Front Immunol. 2024 Jun 26;15:1384361. doi: 10.3389/fimmu.2024.1384361 (PMC11237841; doi:10.3389/fimmu.2024.1384361)
Supplement: Supplementary file 4 [file Table_2.pdf]

Supplementary Table 2. List of Taqman qRT-PCR probes used for validation studies.

Gene	Taqman identifier
GREM1	Hs01879841_s1
TWIST1	Hs00361186_m1
CYP19A1	Hs00903413_m1
CCL2	Hs00234140_m1
S1PR1	Hs00173499_m1
IL6	Hs00985639_m1
