# Supplementary material for: Gravidity influences distinct transcriptional profiles of maternal and fetal placental macrophages at term
Source: Front Immunol. 2024 Jun 26;15:1384361. doi: 10.3389/fimmu.2024.1384361 (PMC11237841; doi:10.3389/fimmu.2024.1384361)
Supplement: Supplementary file 5 [file Table_3.pdf]

Supplementary Table 3. Relative gene expression differences between MIMs and HBCs via RNA-seq and qRT-PCR. Comparisons of fold change (FC) and significance of differentially expressed genes between MIMs and HBCs identified via RNA-seq and qRT-PCR. Pearson's correlation between the two datasets was 0.97. Genes shaded in grey were not defined as significant based on separate significance criteria for the two approaches: qRT-PCR, $p<0.05$; RNA-seq, $p<0.00001$; absolute $\log_2$FC > 2.

**HBCs vs. MIMs**	***TWIST1***	***CYP19A1***	***CCL2***	***S1PR1***	***IL6***	***GREM1***
Log$_2$FC (qRT-PCR)	-4.87991	-4.12415	1.389308	1.10748	1.902728	3.193769
p-value (qRT-PCR)	9.82E-12	8.6E-08	0.000145	0.003528	0.00048	2.23E-07
Log$_2$FC (RNA-seq)	-5.67689	-4.29884	3.58	4.46	3.492038	3.863052
p-value (RNA-seq)	9.5E-12	8.32E-07	4.47E-06	5.76E-07	0.002403	0.003953
