# Supplementary material for: Gravidity influences distinct transcriptional profiles of maternal and fetal placental macrophages at term
Source: Front Immunol. 2024 Jun 26;15:1384361. doi: 10.3389/fimmu.2024.1384361 (PMC11237841; doi:10.3389/fimmu.2024.1384361)
Supplement: Supplementary file 6 [file Table_4.pdf]

Supplementary Table 4: Gene ontology analysis of differentially expressed genes between MIMs and HBCs. Significant biological processes identified via DAVID ($p<0.01$; number changed ≥10). Number of differentially expressed genes higher in MIMs or HBCs are noted. Ratio between the two subsets. P-value regarding significance and list of genes differentially expressed in MIMs or HBCs.

Term	MIM	HBC	Ratio	ALL (p)	MIM_up	HBC_up
**cell motility**	50	49	1.0	5.4E-07	*CGA, PRKCZ, PPP2R3A, PGF, TBX20, WWC1, SLC7A8, GIPC1, POSTN, RDX, CCL28, PTEN, STARD13, PFN2, DAB2, P2RY6, ZNF703, PAK3, CCSAP, GAB1, SEMA3B, NOS3, RAB25, DEPDC1B, RHOD, DCX, NET1, CYP19A1, TWIST1, PARD6B, LURAP1, VAV3, S100P, ARTN, MIEN1, HES1, SDC1, SVBP, RRAS2, OPHN1, HSPB1, WASL, APBB2, ADGRL3, GRB7, EMP2, TNFAIP1, NLRP10, GSTP1, KALRN*	*NRP2, ADCY3, ATP1B1, JAG2, JAG1, SLC7A5, IL10, SLC16A1, S1PR1, UNC5C, RAPGEF2, IL1A, DPP4, MATK, LDB2, PDE4D, LDLRAD4, DDIT4, TNFAIP6, MMP10, CCR7, CCR4, SEMA4C, SRGAP3, NKD1, CCL2, SPOCK2, CXCL3, CSF1, KIT, SRC, HRH1, IL23A, CCL20, ITGAV, TNFRSF18, CD2, ZC3H12A, HSPA5, NFATC2, OLR1, SPHK1, SMAD3, EVL, ADGRG1, PLCG1, ITGA6, ITGA5, ABL2*
**cell migration**	46	41	0.9	5.1E-06	*CGA, PRKCZ, PGF, TBX20, WWC1, SLC7A8, GIPC1, RDX, POSTN, PTEN, CCL28, STARD13, P2RY6, DAB2, PFN2, ZNF703, PAK3, SEMA3B, NOS3, RAB25, DEPDC1B, RHOD, DCX, NET1, TWIST1, CYP19A1, LURAP1, S100P, VAV3, ARTN, MIEN1, HES1, SDC1, SVBP, RRAS2, OPHN1, HSPB1, WASL, APBB2, EMP2, ADGRL3, GRB7, GSTP1, NLRP10, TNFAIP1, KALRN*	*NRP2, ATP1B1, CCL2, CXCL3, CSF1, KIT, SLC7A5, IL10, SRC, HRH1, SLC16A1, S1PR1, IL23A, CCL20, ITGAV, TNFRSF18, CD2, ZC3H12A, HSPA5, NFATC2, RAPGEF2, DPP4, IL1A, MATK, OLR1, SPHK1, SMAD3, PDE4D, EVL, ADGRG1, LDLRAD4, DDIT4, TNFAIP6, CCR7, ITGA6, PLCG1, ITGA5, CCR4, SEMA4C, SRGAP3, ABL2*
**regulation of cell motility**	29	35	1.2	5.6E-07	*CGA, PPP2R3A, PGF, POSTN, RDX, PTEN, CCL28, STARD13, DAB2, PFN2, P2RY6, ZNF703, PAK3, CCSAP, GAB1, SEMA3B, RAB25, RHOD, CYP19A1, TWIST1, PARD6B, MIEN1, SVBP, RRAS2, HSPB1, WASL, GRB7, EMP2, GSTP1*	*NRP2, NKD1, CCL2, SPOCK2, CXCL3, CSF1, JAG2, JAG1, KIT, SRC, S1PR1, IL23A, CCL20, ITGAV, TNFRSF18, ZC3H12A, HSPA5, UNC5C, RAPGEF2, IL1A, SPHK1, LDB2, SMAD3, EVL, ADGRG1, LDLRAD4, MMP10, TNFAIP6, CCR7, ITGA6, PLCG1, ITGA5, SEMA4C, SRGAP3, ABL2*
**regulation of cell migration**	26	32	1.2	4.8E-06	*CGA, PGF, POSTN, RDX, PTEN, CCL28, STARD13, PFN2, DAB2, P2RY6, ZNF703, PAK3, GAB1, SEMA3B, RAB25, RHOD, CYP19A1, PARD6B, MIEN1, SVBP, RRAS2, HSPB1, WASL, GRB7, EMP2, GSTP1*	*NRP2, CCL2, CXCL3, CSF1, JAG2, JAG1, KIT, SRC, S1PR1, IL23A, CCL20, ITGAV, TNFRSF18, ZC3H12A, HSPA5, UNC5C, RAPGEF2, IL1A, SPHK1, LDB2, SMAD3, EVL, ADGRG1, LDLRAD4, TNFAIP6, MMP10, CCR7, ITGA6, PLCG1, ITGA5, SEMA4C, SRGAP3*
**chemotaxis**	14	27	1.9	2.0E-03	*VAV3, STX3, ANK3, PGF, ARTN, OPHN1, HSPB1, SIAH1, SEMA3B, APBB2, CCL28, ETV4, GSTP1, CYP19A1*	*NRP2, CCL2, CXCL3, CSF1, KIT, IL10, HRH1, S1PR1, IL23A, CCL20, BCL11B, ITGAV, CXCR6, UNC5C, DRAXIN, SMAD3, EVL, PDE4D, NCAM1, CCR8, CCR7, CCR4, NTRK1, SEMA4C, SPTBN1, SPTAN1, FEZ1*
**(+) regulation of cell motility**	16	24	1.5	7.4E-06	*CGA, PGF, POSTN, RDX, MIEN1, P2RY6, DAB2, ZNF703, PAK3, RRAS2, HSPB1, SEMA3B, RAB25, RHOD, GRB7, TWIST1*	*NRP2, CCL2, SPOCK2, CSF1, CXCL3, SPHK1, SMAD3, KIT, SRC, TNFAIP6, CCR7, S1PR1, IL23A, ITGA6, PLCG1, CCL20, ITGA5, ITGAV, SEMA4C, TNFRSF18, ZC3H12A, HSPA5, RAPGEF2, IL1A*

(+) regulation of cellular component movement	16	24	1.5	1.4E-05	CGA, PGF, POSTN, RDX, MIEN1, P2RY6, DAB2, ZNF703, PAK3, RRAS2, HSPB1, SEMA3B, RAB25, RHOD, GRB7, TWIST1	NRP2, CCL2, SPOCK2, CSF1, CXCL3, SPHK1, SMAD3, KIT, SRC, TNFAIP6, CCR7, S1PR1, IL23A, ITGA6, PLCG1, CCL20, ITGA5, ITGAV, SEMA4C, TNFRSF18, ZC3H12A, HSPA5, RAPGEF2, IL1A
(+) regulation of cell migration	15	23	1.5	1.9E-05	CGA, PGF, POSTN, RDX, MIEN1, DAB2, P2RY6, ZNF703, PAK3, RRAS2, HSPB1, RAB25, SEMA3B, RHOD, GRB7	NRP2, CCL2, CSF1, CXCL3, SPHK1, SMAD3, KIT, SRC, TNFAIP6, CCR7, S1PR1, IL23A, ITGA6, PLCG1, CCL20, ITGA5, ITGAV, SEMA4C, TNFRSF18, ZC3H12A, HSPA5, RAPGEF2, IL1A
single organismal cell-cell adhesion	20	32	1.6	9.6E-04	PRKCZ, GLDN, NECTIN3, CAMSAP3, RDX, BAD, PAWR, CSRP1, SOD1, CCL28, HES1, IGSF5, ZNF703, PAK3, ANK3, PDE5A, TTYH1, HSPB1, TSTA3, DLG5	CCL2, CD8A, IL6ST, JAG2, KIT, IL7R, TNFRSF4, IL10, SRC, IL23A, ICOS, BCL11B, ITGAV, MAP3K8, CD2, CD6, CD5, DPP4, CD28, CD7, FZD8, ITK, TCF7, CD3E, CTLA4, SMAD3, NLGN2, CCR7, ITGA6, ITGA5, ITGAD, PRNP
(+) regulation of cell adhesion	13	24	1.8	2.6E-05	PRKCZ, STX3, VAV3, CYTH3, BAD, CCL28, HES1, ANK3, PAK3, TGM2, RHOD, EMP2, NET1	CCL2, IL6ST, SPOCK2, CD3E, CSF1, CTLA4, SMAD3, NINJ1, IL7R, ADGRG1, IL10, SRC, CCR7, IL23A, ITGA6, ITGA5, ITGAV, ICOS, MAP3K8, TNFRSF18, CD6, CD5, DPP4, CD28
regulation of cell-cell adhesion	11	18	1.6	8.5E-03	HES1, PRKCZ, ZNF703, PAK3, ANK3, PDE5A, RDX, BAD, PAWR, SOD1, CCL28	CCL2, CD3E, IL6ST, CTLA4, IL7R, IL10, SRC, CCR7, IL23A, ITGA6, ICOS, MAP3K8, CD2, PRNP, CD6, CD5, DPP4, CD28
(+) regulation of cell-cell adhesion	5	16	3.2	8.2E-03	HES1, PRKCZ, PAK3, ANK3, BAD	CCL2, CD3E, IL6ST, CTLA4, IL7R, IL10, SRC, CCR7, IL23A, ITGA6, ICOS, MAP3K8, CD6, CD5, DPP4, CD28
cell-matrix adhesion	9	11	1.2	1.0E-03	PRKCZ, SORBS1, LYPD5, POSTN, RHOD, EMP2, PTEN, CCL28, RASA1	CD96, CCR7, LYPD3, ITGA6, EPDR1, ITGB8, ITGAV, CSF1, SMAD3, NINJ1, SRC
regulation of cell-matrix adhesion	7	5	0.7	2.9E-03	PRKCZ, POSTN, RHOD, EMP2, PTEN, CCL28, RASA1	CCR7, CSF1, SMAD3, NINJ1, SRC
inflammatory response	8	38	4.8	1.7E-03	PRKCZ, SDC1, VAMP8, TGM2, SNAP23, CDO1, GSTP1, CYP19A1	CCL2, LDLR, IL6ST, TNFRSF25, CXCL3, CSF1, DUSP10, NFKB1, AFAP1L2, NFKB2, KIT, TNFRSF4, IL10, CD96, HRH1, IL23A, REL, CCL20, PTGES, TICAM1, CXCR6, TNFRSF18, ZC3H12A, CD6, IL1A, CD28, TNIP3, IRAK2, OLR1, IL1RL1, SPHK1, SMAD3, TNFRSF9, TNFAIP6, CCR7, CCR4, KDM6B, IGFBP4
leukocyte cell-cell adhesion	9	28	3.1	2.6E-03	HES1, PRKCZ, PAK3, PDE5A, TSTA3, BAD, PAWR, SOD1, CCL28	CCL2, CD8A, IL6ST, JAG2, KIT, IL7R, TNFRSF4, IL10, SRC, IL23A, ICOS, BCL11B, MAP3K8, CD2, CD6, CD5, DPP4, CD7, CD28, FZD8, ITK, TCF7, CD3E, CTLA4, SMAD3, CCR7, ITGA5, PRNP
T cell activation	7	27	3.9	3.4E-03	HES1, PRKCZ, PAK3, PDE5A, BAD, PAWR, SOD1	CCL2, CD8A, IL6ST, JAG2, KIT, IL7R, TNFRSF4, IL10, SRC, IL23A, ICOS, BCL11B, MAP3K8, CD2, CD6, CD5, DPP4, CD7, CD28, FZD8, ITK, TCF7, CD3E, CTLA4, SMAD3, CCR7, PRNP

**leukocyte migration**	9	23	2.6	8.2E-04	*VAV3, PGF, SLC7A8, ARTN, WASL, GRB7, CCL28, NLRP10, CYP19A1*	*ATP1B1, CCL2, OLR1, CSF1, CXCL3, PDE4D, KIT, SLC7A5, IL10, SRC, CCR7, SLC16A1, HRH1, S1PR1, IL23A, ITGA6, PLCG1, CCL20, ITGA5, ITGAV, TNFRSF18, CD2, IL1A*
**(+) regulation of cytokine production**	7	24	3.4	2.5E-03	*PRKCZ, LURAP1, HSPB1, POSTN, SOD1, NLRP10, TWIST1*	*IRAK1, CCL2, PANX1, IL6ST, CD3E, IL1RL1, SMAD3, NFKB1, AFAP1L2, PDE4D, NFKB2, IL10, SRC, CCR7, IL23A, CCL20, TICAM1, CD2, HEG1, SPTBN1, CD6, EIF2AK3, IL1A, CD28*
**response to lipopolysaccharide**	2	26	13.0	5.2E-04	*NOS3, GSTP1*	*CSF3, CCL2, TNFRSF25, CXCL3, DUSP10, NFKB1, ABCA1, NFKB2, TNFRSF4, IL10, NOCT, SRC, CD96, CCL20, PTGES, TICAM1, TNFRSF18, ZC3H12A, CD6, TNIP3, IRAK2, IRAK1, PDE4D, TNFRSF9, CCR7, PENK*
**response to molecule of bacterial origin**	2	26	13.0	1.0E-03	*NOS3, GSTP1*	*CSF3, CCL2, TNFRSF25, CXCL3, DUSP10, NFKB1, ABCA1, NFKB2, TNFRSF4, IL10, NOCT, SRC, CD96, CCL20, PTGES, TICAM1, TNFRSF18, ZC3H12A, CD6, TNIP3, IRAK2, IRAK1, PDE4D, TNFRSF9, CCR7, PENK*
**(+) regulation of leukocyte activation**	6	21	3.5	1.7E-03	*HES1, PRKCZ, VAV3, VAMP8, PAK3, BAD*	*CCL2, CD3E, IL1RL1, IL6ST, CTLA4, NECTIN2, IL7R, TNFRSF4, IL10, SRC, CCR7, IL23A, ICOS, TICAM1, MAP3K8, CD2, CD6, NFATC2, CD5, DPP4, CD28*
**(+) regulation of cell activation**	6	21	3.5	2.5E-03	*HES1, PRKCZ, VAV3, VAMP8, PAK3, BAD*	*CCL2, CD3E, IL1RL1, IL6ST, CTLA4, NECTIN2, IL7R, TNFRSF4, IL10, SRC, CCR7, IL23A, ICOS, TICAM1, MAP3K8, CD2, CD6, NFATC2, CD5, DPP4, CD28*
**regulation of leukocyte cell-cell adhesion**	8	17	2.1	7.5E-03	*HES1, PRKCZ, PAK3, PDE5A, BAD, PAWR, SOD1, CCL28*	*CCL2, CD3E, IL6ST, CTLA4, IL7R, IL10, SRC, CCR7, IL23A, ICOS, MAP3K8, CD2, PRNP, CD6, CD5, DPP4, CD28*
**cytokine secretion**	6	11	1.8	3.0E-03	*PRKCZ, POSTN, ANXA4, NLRP10, IL36RN, TWIST1*	*TNFRSF9, CCR7, PANX1, IL1RL1, CD2, ZC3H12A, SPTBN1, ABCA1, IL10, SRC, IL1A*
**regulation of cytokine secretion**	6	10	1.7	3.0E-03	*PRKCZ, POSTN, ANXA4, NLRP10, IL36RN, TWIST1*	*TNFRSF9, CCR7, PANX1, IL1RL1, CD2, ZC3H12A, SPTBN1, IL10, SRC, IL1A*
**cellular response to molecule of bacterial origin**	2	14	7.0	4.1E-03	*NOS3, GSTP1*	*IRAK2, CSF3, IRAK1, CCL2, PDE4D, NFKB1, ABCA1, IL10, SRC, CCL20, TICAM1, ZC3H12A, CD6, TNIP3*
**interleukin-1 production**	2	8	4.0	4.7E-03	*HSPB1, GSTP1*	*CCR7, PANX1, CCL20, SPHK1, SMAD3, ZC3H12A, ABCA1, IL10*

**programmed cell death**	55	53	1.0	3.2E-03	*HTATIP2, MAEL, PAWR, PTEN, DAB2, AES, DYNLL1, PAK3, NOS3, DLG5, NET1, TWIST1, KLLN, SOCS2, TFPT, CRYAB, LGALS13, CECR2, ARHGEF12, STK3, MIEN1, NME6, RFK, LGALS16, SCIN, GADD45G, LGALS14, TFAP2A, HSPB1, SIAH1, GSTP1, TNFAIP1, KALRN, PRKCZ, GULP1, BEX2, UBE2V2, FIS1, AKT1S1, TGM2, PHLDA3, RASA1, TMEM79, VAV3, TBX3, SMAD6, PDK4, RYBP, BAD, SOD1, ANXA4, AKTIP, CFDP1, GRK5, APBB2*	*STIL, IL6ST, LGMN, TNFSF15, JAG2, NFKB1, IL10, TICAM1, MAP3K8, UNC5C, RAPGEF2, IL1A, IRAK1, CD3E, GZMA, DRAXIN, DDIT4, SERPINB9, CTSL, AMIGO2, TNFRSF9, CCR7, CCND2, ERN1, PRNP, EIF2AK3, TRAF1, CCL2, TNFRSF25, HK2, KIT, TNFRSF4, SRC, ITGAV, BCL11B, TNFRSF18, CD2, ZC3H12A, HSPA5, CD5, PHLDA1, CD28, PDK1, IL2RB, TCF7, TP53BP2, SPHK1, CTLA4, SMAD3, ITGA6, ITGA5, NTRK1, DRAM1*
**regulation of programmed cell death**	45	40	0.9	4.4E-03	*PRKCZ, HTATIP2, MAEL, BEX2, UBE2V2, PAWR, PTEN, FIS1, DAB2, AKT1S1, AES, DYNLL1, PAK3, TGM2, NOS3, DLG5, PHLDA3, RASA1, NET1, TWIST1, VAV3, SOCS2, TBX3, CRYAB, SMAD6, PDK4, LGALS13, BAD, ARHGEF12, SOD1, ANXA4, MIEN1, STK3, LGALS16, SCIN, LGALS14, GADD45G, TFAP2A, HSPB1, SIAH1, CFDP1, GRK5, APBB2, GSTP1, KALRN*	*TRAF1, STIL, CCL2, IL6ST, TNFRSF25, LGMN, TNFSF15, NFKB1, KIT, TNFRSF4, IL10, SRC, BCL11B, ITGAV, TNFRSF18, ZC3H12A, HSPA5, UNC5C, RAPGEF2, IL1A, IRAK1, IL2RB, TCF7, TP53BP2, CD3E, GZMA, DRAXIN, SPHK1, CTLA4, SMAD3, SERPINB9, AMIGO2, TNFRSF9, CCR7, ITGA6, CCND2, ITGA5, NTRK1, PRNP, EIF2AK3*
**(-) regulation of cell death**	27	31	1.1	4.8E-03	*PRKCZ, HTATIP2, MAEL, UBE2V2, PTEN, NPAS2, DAB2, TGM2, NOS3, RASA1, TWIST1, TBX3, SOCS2, CRYAB, SMAD6, PDK4, BAD, SOD1, ANXA4, MIEN1, BTBD10, HSPB1, TFAP2A, CFDP1, GRK5, APBB2, GSTP1*	*CSF3, STIL, CCL2, IL6ST, LGMN, NFKB1, KIT, IL10, SRC, REL, BCL11B, ITGAV, TNFRSF18, ZC3H12A, HSPA5, IL1A, IRAK1, TCF7, IL2RB, DRAXIN, SPHK1, SMAD3, AMIGO2, SERPINB9, NPC1, CCR7, ITGA6, CCND2, ITGA5, NTRK1, PRNP*
**(-) regulation of programmed cell death**	25	28	1.1	8.6E-03	*PRKCZ, HTATIP2, MAEL, UBE2V2, PTEN, DAB2, TGM2, NOS3, RASA1, TWIST1, TBX3, SOCS2, CRYAB, SMAD6, PDK4, BAD, SOD1, ANXA4, MIEN1, HSPB1, TFAP2A, CFDP1, GRK5, APBB2, GSTP1*	*STIL, CCL2, IL6ST, LGMN, NFKB1, KIT, IL10, SRC, ITGAV, BCL11B, TNFRSF18, ZC3H12A, HSPA5, IL1A, IRAK1, TCF7, IL2RB, DRAXIN, SPHK1, SMAD3, AMIGO2, SERPINB9, CCR7, ITGA6, CCND2, ITGA5, NTRK1, PRNP*
**regulation of signal transduction**	86	82	1.0	7.6E-06	*SPIN1, TBX20, WWC1, PIP5K1B, POSTN, ARHGAP17, PAWR, PTEN, DAB2, AES, DYNLL1, IFT20, PAK3, GAB1, NOS3, DEPDC1B, RHOD, RNF146, NET1, RS1, TWIST1, PID1, SOCS2, TRIM40, ARHGEF12, STX1B, STK3, CNGA1, MAP4K3, HES1, ATP6V1C2, DACT2, GADD45G, PDE5A, OPHN1, HSPB1, SIAH1, ADAMTS3, EMP2, TNFAIP1, EPS8L1, GSTP1, KALRN, PRKCZ, EID2, PPP2R3A, RAP1GAP, GLIS2, GIPC1, STARD10, RDX, CYTH3, FAM13A, STARD13, RBX1, PSMB5, MTM1, FIS1, AKT1S1, ZNF703, AMER2, SORBS1, SMARCB1, ARHGAP42, TGM2, RHOBTB1, PHLDA3, RASA1, MTMR4, RNF14, FBXO8, DVL3, LURAP1, VAV3, ERH, SMAD6, BAD, CBY1, TAX1BP3, SOD1, TRIM62, CISH, IL36RN, GRK5, GRB7, PLEKHA1*	*NAF1, CD8A, IL6ST, LGMN, EZH2, JAG2, TNFSF15, NFKB1, JAG1, SHE, IL10, SPRY1, SH2D1A, MAP3K8, TICAM1, RAPGEF2, RAMP1, IL1A, TNIP3, IRAK2, IRAK1, TNIK, CD3E, TRABD2A, DRAXIN, PDE4D, PIK3IP1, LDLRAD4, TRAT1, DDIT4, ACVR2A, CCR7, ZFYVE28, SRGAP3, ERN1, SEMA4C, PRNP, PPP1R15B, EIF2AK3, RASD2, PMEPA1, CSF3, TRAF1, NKD1, CCL2, TNFRSF25, CSF1, DUSP10, AFAP1L2, ABCA1, KIT, SESN2, TNFRSF4, SRC, LIF, TSPYL2, IL23A, CCL20, REL, ITGAV, TNFRSF18, ZC3H12A, HSPA5, AXIN2, CD28, FZD8, TP53BP2, IL1RL1, ASXL1, SPHK1, SMAD3, NLGN2, IGSF9B, RGS16, ADGRG1, RGS13, DOT1L, PLCG1, ITGA6, ITGA5, NTRK1, IGFBP4*

**intracellular signal transduction**	81	76	0.9	1.4E-04	*CSH1, RAB5B, GRIP1, TUFT1, MAEL, WWC1, PIP5K1B, ARHGAP17, FGF12, CNOT7, PTEN, ARL5A, PAK3, GAB1, NOS3, RAB6B, RAB25, RHOD, DLG5, DEPDC1B, AKT3, NET1, TWIST1, SOCS2, CRYAB, ARTN, TRIM40, ARHGEF12, NEK11, STK3, MAP4K3, HES1, RRAS2, GADD45G, PDE5A, OPHN1, HSPB1, RAB15, SIAH1, TNFAIP1, EPS8L1, GSTP1, KALRN, PRKCZ, CARHSP1, RAB3B, RAP1GAP, RDX, CYTH3, FAM13A, STARD13, RBX1, PSMB5, MTM1, FIS1, AKT1S1, ARHGAP42, TGM2, RHOBTB1, DCX, PHLDA3, RASA1, FBXO8, RHOBTB3, RAB2A, DVL3, CNKSR1, LURAP1, VAV3, EFS, TEAD3, BAD, TAX1BP3, SOD1, TRIM62, CISH, GH2, ICK, RAB36, APBB2, PLEKHA1*	*ADCY3, NAF1, ATP1B1, CD8A, IL6ST, EZH2, NCS1, TNFSF15, NFKB1, NFKB2, IL10, SPRY1, MAP3K8, TICAM1, RAPGEF2, IL1A, TNIP3, IRAK2, IRAK1, TNIK, CD3E, NCALD, PDE4D, PATJ, PIK3IP1, TRAT1, DDIT4, NCAM1, CCR7, RND1, SRGAP3, ERN1, SEMA4C, PRNP, EIF2AK3, RASD2, CSF3, CCL2, TNFRSF25, CSF1, DUSP10, KIT, ABCA1, SESN2, TNFRSF4, SRC, LIF, HRH1, IL23A, CCL20, REL, PLCH2, ITGAV, TNFRSF18, ZC3H12A, NFATC2, CD28, PDK1, ITK, FZD8, IL2RB, SPSB1, TP53BP2, IL1RL1, SPHK1, DGKH, ITPR3, ADGRG1, RALGDS, RAB33A, DOT1L, PLCG1, NTRK1, SPTBN1, IGFBP4, SPTAN1*
**cell surface receptor signaling pathway**	67	86	1.3	7.3E-04	*CSH1, SPIN1, PGF, TBX20, AP3S1, PAWR, FGF12, PTEN, DAB2, AES, IFT20, PAK3, GAB1, SPG21, SEMA3B, NOS3, DEPDC1B, RNF146, PID1, SOCS2, STX1B, STK3, HES1, ATP6V1C2, SDC1, DACT2, HSPB1, WASL, ADAMTS3, EMP2, GSTP1, KALRN, PRKCZ, EID2, PPP2R3A, GLIS2, GIPC1, RBX1, PSMB5, P2RY6, AKT1S1, ZNF703, AMER2, SORBS1, TGM2, IL1RAPL2, RASA1, MTMR4, DVL3, CNKSR1, VAV3, ERH, SMAD6, PDK4, BAD, CBY1, TAX1BP3, TRIM62, ANXA4, CISH, IL36RN, GH2, ATP6V1E1, GRK5, ADGRL3, GRB7, PLEKHA1*	*NRP2, STIL, CD8A, IL6ST, LGMN, JAG2, TNFSF15, NFKB1, JAG1, SPRY1, CXCR6, TICAM1, UNC5C, RAPGEF2, IL1A, MATK, IRAK2, IRAK1, TNIK, VANGL1, CD3E, TRABD2A, DRAXIN, PDE4D, LDLRAD4, TRAT1, DDIT4, NCAM1, TNFRSF9, ACVR2A, CCR8, CCR7, CCR4, ZFYVE28, SEMA4C, PRNP, EIF2AK3, PMEPA1, KLRC1, CSF3, TRAF1, NKD1, CCL2, TNFRSF25, CXCL3, CSF1, AFAP1L2, KIT, ABCA1, IL7R, TNFRSF4, SRC, LIF, CCL20, ITGB8, ITGAV, CD2, TNFRSF18, ADAMTS10, HSPA5, AXIN2, CD6, NFATC2, CD28, CD7, FZD8, ITK, TCF7, IL2RB, IL1RL1, SPHK1, CTLA4, SMAD3, NLGN2, EVL, IGSF9B, TSPAN18, ADGRG1, ITGA6, PLCG1, ITGA5, NTRK1, SPTBN1, ITGAD, ABL2, IGFBP4*
**regulation of intracellular signal transduction**	48	53	1.1	3.4E-03	*PRKCZ, RAP1GAP, WWC1, PIP5K1B, RDX, ARHGAP17, CYTH3, PTEN, FAM13A, STARD13, FIS1, MTM1, AKT1S1, PAK3, GAB1, ARHGAP42, TGM2, RHOBTB1, RHOD, DEPDC1B, PHLDA3, FBXO8, RASA1, NET1, TWIST1, DVL3, LURAP1, VAV3, SOCS2, TRIM40, BAD, ARHGEF12, SOD1, TRIM62, CISH, STK3, MAP4K3, HES1, PDE5A, GADD45G, OPHN1, HSPB1, SIAH1, GSTP1, EPS8L1, TNFAIP1, PLEKHA1, KALRN*	*NAF1, CD8A, IL6ST, EZH2, TNFSF15, IL10, SPRY1, MAP3K8, TICAM1, RAPGEF2, IL1A, TNIP3, IRAK2, IRAK1, TNIK, CD3E, PDE4D, PIK3IP1, TRAT1, DDIT4, CCR7, SEMA4C, ERN1, SRGAP3, PRNP, EIF2AK3, RASD2, CSF3, CCL2, TNFRSF25, CSF1, DUSP10, ABCA1, KIT, SESN2, TNFRSF4, SRC, LIF, IL23A, REL, CCL20, TNFRSF18, ZC3H12A, CD28, FZD8, TP53BP2, IL1RL1, SPHK1, ADGRG1, DOT1L, PLCG1, NTRK1, IGFBP4*
**(+) regulation of signal transduction**	37	53	1.4	8.2E-04	*PRKCZ, SPIN1, PPP2R3A, WWC1, GIPC1, STARD10, PTEN, PSMB5, FIS1, DAB2, DYNLL1, SORBS1, PAK3, SMARCB1, GAB1, TGM2, DEPDC1B, RNF146, DVL3, LURAP1, ERH, VAV3, SOCS2, BAD, SOD1, TRIM62, STX1B, STK3, MAP4K3, HES1, ATP6V1C2, PDE5A, GADD45G, SIAH1, ADAMTS3, EMP2, GRB7*	*CD8A, IL6ST, EZH2, JAG2, TNFSF15, NFKB1, JAG1, SHE, IL10, SH2D1A, TICAM1, MAP3K8, RAPGEF2, IL1A, IRAK2, IRAK1, TNIK, CD3E, TRAT1, ACVR2A, CCR7, SEMA4C, ERN1, RASD2, CSF3, NKD1, CCL2, TNFRSF25, CSF1, AFAP1L2, KIT, TNFRSF4, SRC, LIF, IL23A, REL, CCL20, TNFRSF18, ZC3H12A, AXIN2, CD28, FZD8, TP53BP2, SPHK1, ASXL1, NLGN2, SMAD3, IGSF9B, ADGRG1, PLCG1, ITGA5, NTRK1, IGFBP4*

(-) regulation of signal transduction	36	35	1.0	1.4E-03	PRKCZ, EID2, PPP2R3A, GLIS2, TBX20, WWC1, PAWR, PTEN, RBX1, PSMB5, MTM1, DAB2, AKT1S1, AES, AMER2, ARHGAP42, NOS3, PHLDA3, RASA1, MTMR4, TWIST1, PID1, DVL3, SOCS2, SMAD6, TRIM40, CBY1, TAX1BP3, STK3, CISH, IL36RN, DACT2, HSPB1, TNFAIP1, GSTP1, PLEKHA1	NAF1, NKD1, IL6ST, LGMN, EZH2, DUSP10, SESN2, IL10, SRC, LIF, SPRY1, ITGAV, TICAM1, ZC3H12A, HSPA5, AXIN2, IL1A, TNIP3, CD3E, IL1RL1, TRABD2A, DRAXIN, ASXL1, SMAD3, PIK3IP1, RGS16, LDLRAD4, DDIT4, RGS13, ITGA6, ZFYVE28, PPP1R15B, PRNP, PMEPA1, IGFBP4
(+) regulation of cell communication	44	54	1.2	4.7E-04	SYT1, PRKCZ, RAB3B, SPIN1, PPP2R3A, ANO1, WWC1, GIPC1, STARD10, PTEN, PSMB5, FIS1, DAB2, DYNLL1, SORBS1, ANK3, SMARCB1, PAK3, GAB1, TGM2, DEPDC1B, RNF146, DVL3, LURAP1, ERH, VAV3, STX3, SOCS2, BAD, SOD1, TRIM62, STX1B, STK3, MAP4K3, HES1, ATP6V1C2, VAMP8, PDE5A, GADD45G, SIAH1, ADAMTS3, EMP2, GRB7, KALRN	CD8A, IL6ST, EZH2, JAG2, TNFSF15, NFKB1, JAG1, SHE, IL10, SH2D1A, MAP3K8, TICAM1, RAPGEF2, IL1A, IRAK2, IRAK1, TNIK, CD3E, TRAT1, ACVR2A, CCR7, SEMA4C, ERN1, RASD2, CSF3, NKD1, CCL2, TNFRSF25, CSF1, AFAP1L2, KIT, TNFRSF4, SRC, LIF, IL23A, REL, CCL20, TNFRSF18, ZC3H12A, AXIN2, CD28, FZD8, TP53BP2, SPHK1, ASXL1, NLGN2, SMAD3, IGSF9B, ITPR3, ADGRG1, PLCG1, ITGA5, NTRK1, IGFBP4
(-) regulation of cell communication	36	36	1.0	6.7E-03	PRKCZ, EID2, PPP2R3A, GLIS2, TBX20, WWC1, PAWR, PTEN, RBX1, PSMB5, MTM1, DAB2, AKT1S1, AES, AMER2, ARHGAP42, NOS3, PHLDA3, RASA1, MTMR4, TWIST1, PID1, DVL3, SOCS2, SMAD6, TRIM40, CBY1, TAX1BP3, STK3, CISH, IL36RN, DACT2, HSPB1, TNFAIP1, GSTP1, PLEKHA1	NAF1, NKD1, IL6ST, LGMN, EZH2, DUSP10, SESN2, IL10, SRC, LIF, SPRY1, ITGAV, TICAM1, ZC3H12A, HSPA5, AXIN2, IL1A, TNIP3, CD3E, IL1RL1, TRABD2A, DRAXIN, ASXL1, SMAD3, ASIC1, PIK3IP1, RGS16, LDLRAD4, DDIT4, RGS13, ITGA6, ZFYVE28, PPP1R15B, PRNP, PMEPA1, IGFBP4
phosphate-containing compound metabolic process	91	86	0.9	2.2E-04	MOCOS, ALPPL2, IMPA2, GDA, WWC1, PIP5K1B, PPCS, SULT2B1, FGF12, PTEN, PIFO, DAB2, DYNLL1, PAK3, GAB1, NOS3, PPP1R14C, PPP1R14B, AKT3, TWIST1, PID1, CEP85, SOCS2, CRYAB, PKIG, TTC7B, ARTN, LGALS13, COQ9, CAMSAP3, PKIB, NEK11, STK3, NME6, MAP4K3, HES1, UMPS, RFK, ADK, PUDP, GADD45G, PGM1, PDE5A, HSPB1, MAPRE3, EMP2, GSTP1, NEK7, KALRN, SSU72, PRKCZ, PPP2R3A, NDUFB7, GNE, SSH3, ABHD5, MMD, ALPP, SMUG1, RBX1, PSMB5, MTM1, PFN2, AKT1S1, REXO2, MLLT1, UCK2, DCX, MTMR7, RASA1, MTMR4, DVL3, PLA2G16, VAV3, ERH, CAP2, NDUFA6, SMAD6, PDK4, PPP1R11, AK3, BPGM, BAD, SOD1, CISH, ICK, CHCHD10, AKTIP, GFPT2, BTBD10, GRK5	ADCY3, NAF1, ATP1B1, CDK17, IL6ST, EZH2, TNFSF15, NFKB1, SPRY1, MAP3K8, RANBP2, AGPAT4, RAPGEF2, CDK15, RAMP1, IL1A, MATK, IRAK2, IRAK1, TNIK, CAMK1G, CD3E, PGAP1, LDB2, PDE4D, PIK3IP1, LDLRAD4, TRAT1, DDIT4, NCAM1, ACVR2A, CCR7, CCND2, ZFYVE28, ERN1, SEMA4C, PRNP, PPP1R15B, EIF2AK3, CLN8, PMEPA1, CSF3, CCL2, TNFRSF25, ENPP3, CSF1, DUSP10, HK2, AFAP1L2, KIT, ABCA1, SESN2, TNFRSF4, SRC, LIF, HRH1, TSPYL2, IL23A, LPCAT1, CCL20, PLCH2, ITGAV, ENO2, TNFRSF18, ZC3H12A, HSPA5, AXIN2, EHD4, CD28, PDK1, FZD8, ITK, IL2RB, MEX3B, SPHK1, SMAD3, DGKH, PLCG1, ITGA6, ITGA5, NTRK1, SPTBN1, ADM2, ABL2, IGFBP4, SPTAN1
regulation of phosphorus metabolic process	42	59	1.4	4.4E-04	PRKCZ, WWC1, MMD, PTEN, PIFO, DAB2, PFN2, AKT1S1, DYNLL1, PAK3, GAB1, MLLT1, NOS3, PPP1R14C, PPP1R14B, TWIST1, PID1, CEP85, DVL3, CAP2, VAV3, SOCS2, SMAD6, PKIG, PPP1R11, PKIB, BPGM, CAMSAP3, BAD, SOD1, STK3, CISH, MAP4K3, HES1, AKTIP, PDE5A, GADD45G, BTBD10, HSPB1, EMP2, MAPRE3, GSTP1	ADCY3, NAF1, IL6ST, EZH2, TNFSF15, SPRY1, MAP3K8, RANBP2, RAPGEF2, RAMP1, IL1A, IRAK2, IRAK1, TNIK, CD3E, LDB2, PDE4D, PIK3IP1, LDLRAD4, DDIT4, ACVR2A, CCR7, CCND2, ZFYVE28, SEMA4C, ERN1, PRNP, PPP1R15B, EIF2AK3, PMEPA1, CSF3, CCL2, TNFRSF25, CSF1, DUSP10, AFAP1L2, ABCA1, KIT, SESN2, TNFRSF4, SRC, LIF, HRH1, IL23A, TSPYL2, CCL20, TNFRSF18, ZC3H12A, HSPA5, AXIN2, EHD4, FZD8, SPHK1, SMAD3, PLCG1, ITGA6, ITGA5, NTRK1, IGFBP4

**(+) regulation of phosphorus metabolic process**	25	43	1.7	2.0E-03	*PRKCZ, MMD, WWC1, PTEN, PIFO, PFN2, DAB2, PAK3, GAB1, NOS3, PID1, DVL3, VAV3, CAP2, BAD, SOD1, STK3, HES1, MAP4K3, AKTIP, GADD45G, PDE5A, BTBD10, MAPRE3, EMP2*	*CSF3, ADCY3, CCL2, IL6ST, TNFRSF25, CSF1, EZH2, TNFSF15, AFAP1L2, ABCA1, KIT, TNFRSF4, SRC, LIF, HRH1, IL23A, CCL20, MAP3K8, TNFRSF18, ZC3H12A, HSPA5, AXIN2, RAPGEF2, RAMP1, IL1A, EHD4, IRAK2, IRAK1, FZD8, TNIK, CD3E, SPHK1, SMAD3, ACVR2A, CCR7, ITGA6, PLCG1, CCND2, ITGA5, NTRK1, SEMA4C, ERN1, IGFBP4*
**cell differentiation**	106	86	0.8	7.9E-03	*SYT1, GLDN, PGF, MAEL, POSTN, ILDR2, TPD52, DAB2, KDF1, IFT20, ANK3, CCSAP, BTBD3, RAB25, EIF2B2, FBXO22, NET1, TWIST1, PID1, CECR2, CHODL, STK3, NEBL, HES1, CRCT1, PDE5A, OPHN1, EMP2, KALRN, KAZN, EID2, PPP2R3A, SSH3, MMD, PAQR7, UBE2V2, ECE2, SMARCB1, NGRN, TMEM79, MAFF, PARD6B, SMAD6, MEA1, BPGM, TEAD3, TRIM62, THSD7A, HOPX, GRK5, PLEKHA1, STEAP4, HLF, HTATIP2, NIF3L1, GRIP1, POU6F2, TBX20, PTEN, BZW2, EFHD1, PAK3, CAMSAP2, SEMA3B, DLG5, ZFAT, KIF2A, TCHH, STX3, KIF17, MPP5, ARTN, NECTIN3, CAMSAP3, STX1B, SDC1, DACT2, CLIC5, RRAS2, GADD45G, SCIN, SIAH1, GSTP1, STON2, PRKCZ, RAP1GAP, FHL1, GLIS2, ABHD5, RDX, CBR1, ZNF703, ZNF750, DCX, ETV5, RASA1, ETV4, ERH, TBX3, EXPH5, BAD, CBY1, SOD1, ANXA4, APBB2, ADGRL3*	*NRP2, FOSL2, CD8A, IL6ST, EZH2, JAG2, NCS1, SLFN5, NFKB1, JAG1, NFKB2, SLC7A5, IL10, NOCT, S1PR1, PBXIP1, SNPH, SLC9B2, UNC5C, RAPGEF2, IL1A, MATK, TNIK, CD3E, DRAXIN, MMP19, ZHX2, NECTIN2, PDE4D, LDLRAD4, DDIT4, NCAM1, ACVR2A, CCR7, RND1, CCR4, SEMA4C, EIF2AK3, KDM6B, CSF3, PHLDB1, NKD1, CCL2, CSF1, DUSP10, MYEF2, KIT, ABCA1, IL7R, SRC, MLF1, ITM2A, LIF, LAMB3, IL23A, ITGAV, BCL11B, CD2, ZC3H12A, HEG1, COL6A1, NFATC2, AXIN2, ETV3, CD28, MAF, FZD8, ITK, TCF7, ASXL1, TMEM120B, CTLA4, CENPF, SMAD3, EVL, ADGRG1, WHRN, ABCG1, PENK, ITGA6, ITGA5, NTRK1, SPTBN1, ABL2, SPTAN1, FEZ1*
**system development**	127	98	0.8	3.9E-03	*SYT1, CGA, GDA, GLDN, PGF, TUFT1, POSTN, ILDR2, FGF12, MYLIP, TPD52, DAB2, KDF1, IFT20, ANK3, CCSAP, BTBD3, GAB1, TRIM45, PMS2, EIF2B2, FBXO22, TWIST1, CRYAB, CECR2, CHODL, CDO1, STK3, NEBL, HES1, CRCT1, TFAP2A, HSPB1, OPHN1, EMP2, KALRN, KAZN, EID2, PPP2R3A, SSH3, MMD, UBE2V2, PSMB5, ECE2, SMARCB1, NGRN, IL1RAPL2, TMEM79, CYP19A1, DVL3, MAFF, PARD6B, VAV3, CRIP2, SMAD6, MEA1, BPGM, TEAD3, TRIM62, LIN7A, THSD7A, HOPX, PLEKHA1, BMI1, HLF, HTATIP2, NIF3L1, GRIP1, POU6F2, TBX20, PTEN, BZW2, EFHD1, AES, DYNLL1, PAK3, PCP4, CAMSAP2, NOS3, SEMA3B, STRA6, DLG5, ZFAT, COX17, RS1, KIF2A, TCHH, STX3, MAN1A2, KIF17, MPP5, ARTN, CAMSAP3, NECTIN3, STX1B, SDC1, UMPS, DACT2, CLIC5, SCIN, SIAH1, GSTP1, STON2, PRKCZ, GLRX5, RAP1GAP, HSD17B1, FHL1, GLIS2, STARD13, MTM1, NPAS2, ZNF703, TGM2, PAFAH1B2, DCX, ETV5, ETV4, RASA1, TBX3, PLAC1, EXPH5, CBY1, BAD, SOD1, APBB2, ADGRL3*	*NRP2, STIL, FOSL2, CD8A, IL6ST, EZH2, JAG2, NCS1, JAG1, NFKB2, SLC7A5, IL10, SPRY1, S1PR1, SNPH, SLC9B2, UNC5C, RAPGEF2, RAMP1, IL1A, TNIK, VANGL1, CD3E, PGAP1, DRAXIN, MMP19, ZHX2, LDB2, LDLRAD4, DDIT4, NCAM1, AMIGO2, ACVR2A, CCR7, RND1, XIRP1, CCR4, SEMA4C, EIF2AK3, CLN8, KDM6B, CSF3, ABLIM1, NKD1, CCL2, SPOCK2, CSF1, HK2, DUSP10, NINJ1, MYEF2, KIT, IL7R, SRC, MLF1, ITM2A, LIF, IL23A, LPCAT1, ITGB8, ICOS, ITGAV, BCL11B, CD2, ZC3H12A, HEG1, HSPA5, AXIN2, BCOR, CD28, MAF, FZD8, ITK, TCF7, ASXL1, SPHK1, CTLA4, CENPF, SMAD3, NLGN2, EVL, IGSF9B, ADGRG1, WHRN, PENK, ITGA6, PLCG1, ITGA5, BNC2, NTRK1, MAMLD1, SPTBN1, ADM2, TMEM41B, ABL2, IGFBP4, SPTAN1, FEZ1*

**nervous system development**	67	55	0.8	5.7E-03	*SYT1, GDA, GLDN, NIF3L1, GRIP1, POU6F2, TBX20, POSTN, MYLIP, FGF12, PTEN, BZW2, EFHD1, DYNLL1, IFT20, PCP4, PAK3, ANK3, CAMSAP2, CCSAP, BTBD3, SEMA3B, DLG5, EIF2B2, COX17, KIF2A, TWIST1, STX3, KIF17, MPP5, ARTN, CHODL, CECR2, CAMSAP3, STX1B, STK3, HES1, CLIC5, TFAP2A, OPHN1, SIAH1, GSTP1, KALRN, PRKCZ, PPP2R3A, RAP1GAP, SSH3, GLIS2, MMD, UBE2V2, NPAS2, ECE2, SMARCB1, NGRN, PAFAH1B2, DCX, IL1RAPL2, ETV5, ETV4, PARD6B, DVL3, TBX3, TEAD3, BAD, SOD1, APBB2, ADGRL3*	*NRP2, STIL, IL6ST, EZH2, JAG2, NCS1, JAG1, SLC7A5, S1PR1, SNPH, UNC5C, RAPGEF2, TNIK, DRAXIN, PGAP1, ZHX2, DDIT4, NCAM1, AMIGO2, RND1, CCR4, SEMA4C, CLN8, EIF2AK3, KDM6B, NKD1, CCL2, SPOCK2, CSF1, DUSP10, MYEF2, NINJ1, KIT, SRC, ITM2A, LIF, BCL11B, ZC3H12A, HSPA5, FZD8, TCF7, SPHK1, NLGN2, CENPF, EVL, IGSF9B, WHRN, ADGRG1, PENK, NTRK1, SPTBN1, TMEM41B, ABL2, FEZ1, SPTAN1*
**(+) regulation of synaptic transmission**	7	5	0.7	9.6E-03	*PRKCZ, SYT1, RAB3B, STX3, STX1B, PTEN, KALRN*	*CCL2, NTRK1, NLGN2, IGSF9B, ITPR3*
**single-organism organelle organization**	68	28	0.4	3.1E-03	*SYT1, CHMP5, PEX3, MYLIP, PAWR, TMEM141, AUNIP, ANKRD53, DYNLL1, IFT20, PAK3, ANK3, CAMSAP2, CCSAP, RHOD, KIF2A, PID1, CEP85, STX3, DSN1, CRYAB, VTI1B, CAMSAP3, SPIRE2, STX1B, NEBL, VAMP8, SCIN, OPHN1, WASL, EMP2, TNFAIP1, MAP7D3, NEK7, TPPP3, PRKCZ, STX7, CRIPT, SSH3, BET1, RDX, CHMP2B, FIS1, MTM1, PFN2, PEX19, SORBS1, FIGN, C10ORF90, SKA2, SNAP23, RASA1, SYNPO, CAPN6, GABARAPL1, LURAP1, VAV3, CAP2, MSRB1, BAD, CBY1, SOD1, MSRB2, ICK, SLAIN2, HEBP2, CCDC113, KCTD17*	*ABLIM1, CSF3, STIL, PRC1, HK2, KIT, TPM2, SRC, SPRY1, SLC16A1, S1PR1, STARD9, TBC1D4, RANBP2, AXIN2, TNIK, TP53BP2, SMAD3, CENPF, NECTIN2, EVL, CCR7, RND1, XIRP1, SPAG5, SPTBN1, ABL2, SPTAN1*
**cytoskeleton organization**	47	25	0.5	3.0E-03	*TPPP3, PRKCZ, CHMP5, CRIPT, SSH3, RDX, PAWR, MYLIP, TMEM141, AUNIP, CHMP2B, MTM1, PFN2, ANKRD53, DYNLL1, SORBS1, FIGN, ANK3, PAK3, CCSAP, CAMSAP2, SKA2, RHOD, RASA1, KIF2A, SYNPO, CAPN6, CEP85, LURAP1, CAP2, CRYAB, MSRB1, CECR2, CAMSAP3, SPIRE2, SOD1, MSRB2, NEBL, SLAIN2, SCIN, TUBAL3, OPHN1, WASL, EMP2, MAP7D3, TNFAIP1, NEK7*	*ABLIM1, CSF3, PHLDB1, STIL, CCL2, PRC1, KIT, TPM2, SRC, SPRY1, SLC16A1, S1PR1, STARD9, TUBB6, TNIK, SMAD3, NECTIN2, EVL, CCR7, RND1, XIRP1, SPAG5, SPTBN1, ABL2, SPTAN1*
**plasma membrane organization**	20	6	0.3	7.1E-04	*PID1, PRKCZ, STX3, STX7, TTC7B, MPP5, VTI1B, LYPLA1, RDX, SOD1, PTEN, DAB2, SORBS1, PACSIN3, VAMP8, ANK3, IFT20, WASL, EMP2, KALRN*	*ATP1B1, TNIK, GOLGA7B, SPTBN1, RAPGEF2, RAMP1*
**regulation of plasma membrane organization**	10	1	0.1	1.8E-03	*PID1, DAB2, STX7, STX3, SORBS1, VAMP8, VTI1B, LYPLA1, WASL, KALRN*	*SPTBN1*
