# Supplementary material for: Gravidity influences distinct transcriptional profiles of maternal and fetal placental macrophages at term
Source: Front Immunol. 2024 Jun 26;15:1384361. doi: 10.3389/fimmu.2024.1384361 (PMC11237841; doi:10.3389/fimmu.2024.1384361)
Supplement: Supplementary file 7 [file Table_5.pdf]

Supplementary Table 5. Differential expression of M1/M2 markers in MIMs vs. HBCs. A panel of 19 pro-inflammatory (M1) and 12 anti-inflammatory (M2) markers were identified based on a broad literature review. Out of this panel, 15 genes were observed to be differentially expressed between MIMs and HBCs (average expression>0, p<0.01 and absolute $log_2FC > 1$; please note less conservative criteria than unbiased analysis). Abbreviations include: Log_2 average counts in MIMs (MIM_Ct) or HBCs (HBC_Ct); fold change difference between MIMs and HBCs (FC, log_2); and significance (p-value). Red color indicates significance.

Gene	Function	Status	MIM_Ct	HBC_Ct	MIMs/HBCs log_2FC	p-value
CCL2	**Cytokine**	**M1**	**1.7**	**5.3**	**-3.6**	**6.3E-06**
CD80	**Membrane protein**	**M1**	**0.4**	**3.7**	**-3.3**	**7.4E-05**
CD86	Membrane protein	M1	4.8	6.7	-1.8	2.8E-02
CXCL9	Chemokine	M1	2.8	4.7	-1.8	1.3E-01
CXCL10	Chemokine	M1	2.4	4.3	-1.7	1.1E-01
CXCL11	Chemokine	M1	-0.4	1.6	-2.0	4.8E-02
ICAM3 (CD50)	Intercellular adhesion molecule	M1	5.4	6.3	-0.9	2.4E-01
IDO1	**Enzyme**	**M1**	**1.1**	**4.5**	**-3.3**	**3.7E-03**
IDO2	Enzyme	M1	-1.5	-0.2	-1.4	1.9E-02
IL1A	**Cytokine**	**M1**	**2.3**	**8.4**	**-5.9**	**4.4E-06**
IL1B	**Cytokine**	**M1**	**9.1**	**12.4**	**-3.4**	**1.6E-03**
IL6	**Cytokine**	**M1**	**4.2**	**7.8**	**-3.5**	**2.4E-03**
IL12A	Cytokine	M1	-1.8	0.0	-1.7	1.5E-02
IL12B	Cytokine	M1	-0.4	-1.3	0.9	1.5E-01
IL23A	**Cytokine**	**M1**	**0.3**	**6.5**	**-6.2**	**2.3E-06**
IRF5	Transcription factor	M1	4.6	4.9	-0.3	6.6E-01
TLR2	Toll-like receptor	M1	6.0	8.1	-2.1	1.1E-02
TLR4	Toll-like receptor	M1	5.6	7.7	-2.1	1.1E-02
TNF	Cytokine	M1	6.2	8.0	-1.8	4.2E-02
CCL22	**Chemokine**	**M2**	**0.2**	**4.3**	**-4.0**	**4.9E-04**
CCL24	Chemokine	M2	-2.3	-1.9	-0.3	7.3E-01
CD23 (FCER2)	Membrane protein	M2	3.1	2.4	0.8	3.5E-01
CD163	**Scavenger receptor**	**M2**	**6.7**	**8.9**	**-2.2**	**1.3E-03**
CD209	**Membrane receptor**	**M2**	**1.6**	**4.3**	**-2.8**	**2.3E-04**
IL10	**Cytokine**	**M2**	**2.1**	**5.8**	**-3.7**	**7.1E-06**
TGFB1	Growth Factor	M2	8.9	9.2	-0.3	3.0E-01
TGM2	**Enzyme**	**M2**	**11.8**	**8.1**	**3.7**	**1.2E-07**
TREM2	**Membrane receptor**	**M2**	**2.9**	**5.7**	**-2.7**	**5.7E-03**
VEGFA	**Growth Factor**	**M2**	**5.5**	**9.7**	**-4.2**	**1.0E-05**
VEGFB	**Growth Factor**	**M2**	**6.1**	**5.0**	**1.0**	**2.0E-03**
VEGFC	Growth Factor	M2	-3.2	-1.1	-2.1	8.5E-02
