# Supplementary material for: Gravidity influences distinct transcriptional profiles of maternal and fetal placental macrophages at term
Source: Front Immunol. 2024 Jun 26;15:1384361. doi: 10.3389/fimmu.2024.1384361 (PMC11237841; doi:10.3389/fimmu.2024.1384361)
Supplement: Supplementary file 8 [file Table_6.pdf]

Supplementary Table 6: Gene ontology analysis of genes influenced by gravidity in MIMs or HBCs. Significant biological processes influenced by gravid status in MIMs or HBCs identified via DAVID. Criteria: p<0.01 in either MIMs or HBCs and minimum differentially expressed genes ≥ 10.

**Term**	**MIMs (p)**	**MIMs (# genes)**	**HBCs (p)**	**HBCs (# genes)**	**MIMs (genes)**	**HBCs (genes)**
**system development**	0.014	47	0.003	45	*E2F1, PALM, TNF, TCAP, POMK, E2F7, EZH2, DUOX2, CABP4, CKB, FAM83D, PACSIN1, KRT27, FRMD7, BOK, NRARP, RASGRP1, TNR, IL1B, TNFRSF19, SIK1, THBS1, FOSL1, KNDC1, IL1A, HELLS, ICAM1, AR, IL6, MKI67, CHAC1, IL1RL2, MACROD2, NR4A3, UPK3A, NLRP3, MSC, DHRS2, GNGT1, MYO18B, KRT17, WDR62, IFNB1, HES4, F3, SPTBN2, MYLK*	*CSF3, CSF2, STOX1, CPLX2, SLC38A3, WNT5B, HNF1A, RBP1, IGF2BP1, LRRC17, DDR2, ADCYAP1, VCAM1, SLC1A2, HOXA3, CYP27B1, HEY1, CDNF, ROBO1, IFNG, MT1G, PLAG1, UCN, PTPRD, GNAO1, RBM20, MACROD2, RPGRIP1, UPK3A, COL5A3, HMGA2, SIGLEC15, MSC, TNNI1, SSTR2, RND1, BVES, NUPR1, EPGN, MEOX1, TENM2, LRP6, MYRF, PLA2G2D, ADAMTS4*
**animal organ development**	0.012	37	0.003	36	*E2F1, TNF, TCAP, POMK, E2F7, EZH2, DUOX2, CABP4, CKB, FAM83D, KRT27, BOK, NRARP, RASGRP1, TNR, TNFRSF19, SIK1, FOSL1, KNDC1, ICAM1, AR, IL6, MKI67, IL1RL2, MACROD2, NR4A3, UPK3A, NLRP3, MSC, GNGT1, DHRS2, MYO18B, KRT17, WDR62, IFNB1, SPTBN2, MYLK*	*CSF3, CSF2, STOX1, SLC38A3, WNT5B, HNF1A, RBP1, IGF2BP1, LRRC17, DDR2, ADCYAP1, VCAM1, SLC1A2, HOXA3, CYP27B1, HEY1, ROBO1, IFNG, MT1G, PLAG1, GNAO1, RBM20, MACROD2, RPGRIP1, UPK3A, HMGA2, COL5A3, MSC, SIGLEC15, TNNI1, SSTR2, BVES, NUPR1, MEOX1, LRP6, PLA2G2D*
**regulation of signal transduction**	0.001	38	0.098	26	*E2F1, TRAF1, PALM, TNF, EZH2, SPINK1, FAM83D, KIF7, JSRP1, TRIM68, CCL20, FRMD7, BOK, NRARP, RASGRP1, APOC3, TNFRSF19, IL1B, GNG4, THBS1, HELLS, IL1A, ICAM1, AR, IL6, IL1RL1, CHAC1, TPX2, RPH3AL, BIRC5, NLRP3, GNGT1, LYNX1, IFNB1, F3, DUSP8, GADD45A, PTGDR2*	*CSF3, CSF2, STOX1, FGFR4, HNF1A, WNT5B, NKD2, ARHGEF26, TNFSF15, BDKRB2, ADCYAP1, AKR1C2, CYP27B1, HEY1, JSRP1, ROBO1, IFNG, ARC, DGKI, HMGA2, ARHGAP32, CCL13, EPGN, LRP6, RWDD3, RASD2*
**intracellular signal transduction**	0.005	34	0.172	24	*E2F1, TNF, E2F7, EZH2, SPINK1, FAM83D, CCL20, FRMD7, JSRP1, GRIN2C, BOK, RASGRP1, APOC3, TNFRSF19, IL1B, SIK1, THBS1, KNDC1, HELLS, IL1A, ICAM1, AR, IL6, CHAC1, IL1RL1, TPX2, CDC25C, NLRP3, IFNB1, F3, SPTBN2, PTGDR2, DUSP8, GADD45A*	*CSF3, CSF2, STOX1, FGFR4, ARHGEF26, TNFSF15, BDKRB2, DGKI, HMGA2, ADCYAP1, VCAM1, TIFAB, AKR1C2, ARHGAP32, CCL13, RND1, NUPR1, JSRP1, EPGN, ROBO1, TENM2, IFNG, SELE, RASD2*

**regulation of intracellular signal transduction**	0.004	25	0.095	18	*TNF, EZH2, SPINK1, FAM83D, FRMD7, CCL20, JSRP1, BOK, RASGRP1, APOC3, TNFRSF19, IL1B, THBS1, HELLS, IL1A, ICAM1, IL6, AR, IL1RL1, TPX2, NLRP3, IFNB1, F3, DUSP8, GADD45A*	*CSF3, CSF2, STOX1, FGFR4, ARHGEF26, TNFSF15, DGKI, BDKRB2, HMGA2, ADCYAP1, AKR1C2, ARHGAP32, CCL13, JSRP1, EPGN, ROBO1, IFNG, RASD2*
**negative regulation of cell communication**	0.006	19	0.459	10	*ICAM1, PALM, AR, IL6, TNF, CHAC1, IL1RL1, EZH2, RPH3AL, SPINK1, NLRP3, KIF7, NRARP, TNR, IL1B, THBS1, DUSP8, HELLS, IL1A*	*CSF2, UCN, WNT5B, NKD2, HEY1, ROBO1, LRP6, ASIC1, BDKRB2, HMGA2*
**negative regulation of signal transduction**	0.006	18	0.657	8	*ICAM1, PALM, AR, IL6, TNF, CHAC1, IL1RL1, EZH2, RPH3AL, SPINK1, NLRP3, KIF7, NRARP, IL1B, THBS1, DUSP8, IL1A, HELLS*	*CSF2, WNT5B, NKD2, HEY1, ROBO1, LRP6, BDKRB2, HMGA2*
**signal transduction by protein phosphorylation**	0.004	16	0.873	5	*ICAM1, AR, IL6, TNF, EZH2, FAM83D, CCL20, GRIN2C, RASGRP1, SPTBN2, TNFRSF19, IL1B, THBS1, GADD45A, DUSP8, IL1A*	*CSF2, CCL13, FGFR4, EPGN, ADCYAP1*
**cell-cell signaling**	0.675	12	0.010	20	*AR, IL6, TNF, CCL20, NRARP, GRIN2C, TNR, RPH3AL, IL1B, BIRC5, PTGDR2, KCNJ3*	*ARC, PTPRD, UCN, CPLX2, NKD2, WNT5B, HNF1A, GNAO1, SLC6A12, DGKI, ASIC1, HMGA2, ADCYAP1, SSTR2, CCL13, SLC1A2, KCNQ3, IFNG, LRP6, RASD2*
**regulation of protein secretion**	0.001	11	0.940	2	*IL6, TNF, IL1RL1, RASGRP1, RPH3AL, IL1B, BIRC5, NLRP3, NLRP10, IL1A, SCAMP5*	*HNF1A, IFNG*
**protein secretion**	0.004	11	0.966	2	*IL6, TNF, IL1RL1, RASGRP1, RPH3AL, IL1B, BIRC5, NLRP3, NLRP10, IL1A, SCAMP5*	*HNF1A, IFNG*
**positive regulation of secretion**	0.002	10	0.460	4	*IL6, TNF, IL1RL1, RASGRP1, RPH3AL, IL1B, NLRP3, NLRP10, IL1A, SCAMP5*	*UCN, P2RY2, IFNG, ADCYAP1*
**positive regulation of secretion by cell**	0.001	10	0.676	3	*IL6, TNF, IL1RL1, RASGRP1, RPH3AL, IL1B, NLRP3, NLRP10, IL1A, SCAMP5*	*UCN, IFNG, ADCYAP1*
**positive regulation of protein secretion**	0.000	10	1.000	1	*IL6, TNF, IL1RL1, RASGRP1, RPH3AL, IL1B, NLRP3, NLRP10, IL1A, SCAMP5*	*IFNG*
**synaptic signaling**	0.865	4	0.000	14	*GRIN2C, TNR, RPH3AL, PTGDR2*	*ARC, UCN, CPLX2, PTPRD, SLC6A12, ASIC1, DGKI, ADCYAP1, SLC1A2, SSTR2, KCNQ3, IFNG, LRP6, RASD2*

**regulation of protein metabolic process**	0.008	32	0.412	20	*TNF, EZH2, SPINK1, PTTG1, FAM83D, CCL20, GRIN2C, BOK, RASGRP1, TNFRSF19, IL1B, THBS1, KNDC1, IL1A, RNF144A, ICAM1, AR, IL6, ASTL, CHAC1, PTTG3P, TPX2, BIRC5, CDC25C, NLRP3, KRT17, IFNB1, F3, BUB1B, WFDC1, DUSP8, GADD45A*	*CSF3, CSF2, STOX1, FGFR4, UCN, NKD2, TNFSF15, IGF2BP1, BDKRB2, AZIN2, DDR2, ADCYAP1, CCL13, NUPR1, EPGN, ROBO1, IFNG, LRP6, RWDD3, RASD2*
**regulation of cellular protein metabolic process**	0.005	31	0.392	19	*TNF, EZH2, SPINK1, PTTG1, FAM83D, CCL20, BOK, RASGRP1, TNFRSF19, IL1B, THBS1, KNDC1, IL1A, RNF144A, ICAM1, AR, IL6, ASTL, CHAC1, PTTG3P, TPX2, BIRC5, CDC25C, NLRP3, KRT17, IFNB1, F3, BUB1B, WFDC1, DUSP8, GADD45A*	*CSF3, CSF2, STOX1, FGFR4, UCN, NKD2, TNFSF15, IGF2BP1, BDKRB2, DDR2, ADCYAP1, CCL13, NUPR1, EPGN, ROBO1, IFNG, LRP6, RWDD3, RASD2*
**positive regulation of cellular protein metabolic process**	0.004	22	0.075	16	*RNF144A, ICAM1, AR, IL6, TNF, ASTL, EZH2, TPX2, NLRP3, CCL20, KRT17, BOK, IFNB1, F3, RASGRP1, TNFRSF19, IL1B, BUB1B, THBS1, GADD45A, KNDC1, IL1A*	*CSF3, CSF2, STOX1, FGFR4, UCN, NKD2, TNFSF15, DDR2, ADCYAP1, CCL13, NUPR1, EPGN, ROBO1, IFNG, RWDD3, RASD2*
**positive regulation of protein metabolic process**	0.008	22	0.111	16	*RNF144A, ICAM1, AR, IL6, TNF, ASTL, EZH2, TPX2, NLRP3, CCL20, KRT17, BOK, IFNB1, F3, RASGRP1, TNFRSF19, IL1B, BUB1B, THBS1, GADD45A, KNDC1, IL1A*	*CSF3, CSF2, STOX1, FGFR4, UCN, NKD2, TNFSF15, DDR2, ADCYAP1, CCL13, NUPR1, EPGN, ROBO1, IFNG, RWDD3, RASD2*
**MAPK cascade**	0.003	16	0.852	5	*ICAM1, AR, IL6, TNF, EZH2, FAM83D, CCL20, GRIN2C, RASGRP1, SPTBN2, TNFRSF19, IL1B, THBS1, GADD45A, DUSP8, IL1A*	*CSF2, CCL13, FGFR4, EPGN, ADCYAP1*
**programmed cell death**	0.003	27	0.856	11	*TRAF1, LGALS17A, E2F1, TNF, BOK, TNFRSF19, IL1B, THBS1, SIK1, FOSL1, HELLS, IL1A, ICAM1, AR, IL6, CHAC1, TPX2, BIRC5, NR4A3, MCM2, NLRP3, GNGT1, DHRS2, IFNB1, F3, BUB1B, GADD45A*	*KCNMA1, CSF2, UCN, NUPR1, ROBO1, IFNG, TNFSF15, LRP6, BDKRB2, HMGA2, ADCYAP1*
**regulation of programmed cell death**	0.008	21	0.546	11	*E2F1, TRAF1, LGALS17A, ICAM1, AR, IL6, TNF, BIRC5, NR4A3, NLRP3, DHRS2, IFNB1, BOK, F3, IL1B, THBS1, SIK1, GADD45A, FOSL1, HELLS, IL1A*	*KCNMA1, CSF2, UCN, NUPR1, ROBO1, IFNG, TNFSF15, LRP6, BDKRB2, HMGA2, ADCYAP1*
**positive regulation of programmed cell death**	0.001	14	0.387	6	*LGALS17A, E2F1, ICAM1, IL6, TNF, NR4A3, NLRP3, IFNB1, BOK, F3, THBS1, SIK1, GADD45A, FOSL1*	*KCNMA1, NUPR1, ROBO1, IFNG, TNFSF15, HMGA2*

**positive regulation of cell death**	0.001	14	0.429	6	*LGALS17A, E2F1, ICAM1, IL6, TNF, NR4A3, NLRP3, IFNB1, BOK, F3, THBS1, SIK1, GADD45A, FOSL1*	*KCNMA1, NUPR1, ROBO1, IFNG, TNFSF15, HMGA2*
**apoptotic signaling pathway**	0.007	12	0.785	4	*TRAF1, E2F1, ICAM1, AR, TNF, BOK, IFNB1, CHAC1, IL1B, THBS1, HELLS, IL1A*	*CSF2, NUPR1, IFNG, BDKRB2*
**regulation of apoptotic signaling pathway**	0.001	11	0.935	2	*TRAF1, E2F1, ICAM1, AR, TNF, BOK, IFNB1, IL1B, THBS1, HELLS, IL1A*	*CSF2, BDKRB2*
**cell cycle process**	0.000	26	0.989	5	*E2F1, E2F7, EZH2, PTTG1, GPR3, FAM83D, OIP5, NCAPG, IL1B, THBS1, TUBB1, HELLS, IL1A, MKI67, PTTG3P, TPX2, NDC80, BIRC5, GPR132, MCM2, CDC25C, FSD1, WDR62, SPAG5, BUB1B, GADD45A*	*STOX1, CYP27B1, EPGN, IFNG, HMGA2*
**mitotic cell cycle**	0.000	22	0.993	3	*E2F1, MKI67, E2F7, EZH2, TPX2, BIRC5, NDC80, GPR132, PTTG1, MCM2, CDC25C, FSD1, FAM83D, OIP5, NCAPG, SPAG5, WDR62, BUB1B, IL1B, GADD45A, HELLS, IL1A*	*STOX1, EPGN, HMGA2*
**regulation of cell cycle process**	0.000	16	0.927	3	*E2F1, MKI67, E2F7, EZH2, TPX2, NDC80, BIRC5, GPR132, CDC25C, GPR3, FAM83D, SPAG5, IL1B, BUB1B, GADD45A, IL1A*	*STOX1, EPGN, HMGA2*
**regulation of mitotic cell cycle**	0.000	14	0.866	3	*E2F1, MKI67, E2F7, EZH2, TPX2, NDC80, GPR132, BIRC5, CDC25C, IL1B, BUB1B, SIK1, GADD45A, IL1A*	*STOX1, EPGN, HMGA2*
**mitotic nuclear division**	0.000	14	0.813	3	*TPX2, BIRC5, NDC80, PTTG1, CDC25C, FSD1, FAM83D, NCAPG, SPAG5, OIP5, IL1B, BUB1B, IL1A, HELLS*	*STOX1, EPGN, HMGA2*
**positive regulation of cell proliferation**	0.141	11	0.003	15	*E2F1, AR, IL6, TNF, NRARP, F3, IL1B, BIRC5, NR4A3, THBS1, FOSL1*	*PLAG1, CSF3, CSF2, STOX1, FGFR4, HMGA2, DDR2, ADCYAP1, ALDH3A1, VCAM1, AKR1C2, HOXA3, EPGN, IFNG, MPL*
**mitotic cell cycle phase transition**	0.008	11	0.975	2	*E2F1, E2F7, EZH2, TPX2, BUB1B, GPR132, BIRC5, NDC80, MCM2, CDC25C, GADD45A*	*STOX1, HMGA2*
**positive regulation of cell cycle**	0.000	11	0.431	4	*FAM83D, E2F1, E2F7, IL1B, BIRC5, NDC80, NR4A3, CDC25C, GADD45A, FOSL1, IL1A*	*STOX1, EPGN, LRP6, HMGA2*

**regulation of cell cycle phase transition**	0.002	10	0.910	2	*FAM83D, E2F1, E2F7, EZH2, BUB1B, GPR132, BIRC5, NDC80, CDC25C, GADD45A*	*STOX1, HMGA2*
**single-organism organelle organization**	0.002	25	0.997	5	*E2F1, ICAM1, TNF, TCAP, MYO1B, MAP1A, TPX2, RPH3AL, NDC80, BIRC5, SYNPO2, PACSIN1, PKP1, FRMD7, KRT17, BOK, WDR62, NCAPG, SPAG5, CCDC114, SPTBN2, BUB1B, TUBB1, GADD45A, KATNAL2*	*CSF3, NKD2, RND1, CCDC151, MNS1*
**cytoskeleton organization**	0.000	22	0.827	7	*ICAM1, PALM, TNF, TCAP, MYO1B, MAP1A, TPX2, NDC80, BIRC5, SYNPO2, PACSIN1, PKP1, FRMD7, KRT17, ZNF135, SPAG5, WDR62, CCDC114, SPTBN2, TUBB1, GADD45A, KATNAL2*	*CSF3, ARC, CCL13, RND1, ZNF135, CCDC151, SIGLEC15*
**organelle fission**	0.000	17	0.938	3	*MKI67, PTTG3P, TPX2, NDC80, BIRC5, PTTG1, CDC25C, GPR3, FSD1, FAM83D, NCAPG, SPAG5, OIP5, IL1B, BUB1B, IL1A, HELLS*	*STOX1, EPGN, HMGA2*
**microtubule cytoskeleton organization**	0.007	10	1.000	1	*WDR62, SPAG5, CCDC114, MAP1A, TPX2, BIRC5, NDC80, TUBB1, KATNAL2, GADD45A*	*CCDC151*
**response to cytokine**	0.000	20	0.114	10	*TRAF1, ICAM1, IL6, TNF, IL1RL1, IL1RL2, CXCL3, DUOX2, CXCL2, MCM2, CCRL2, CCL20, TRIM68, IFNB1, F3, TNFRSF19, IL1B, THBS1, FOSL1, IL1A*	*VCAM1, CSF3, CCL13, GNAO1, CYP27B1, ROBO1, IFNG, TNFSF15, MPL, SELE*
**inflammatory response**	0.000	16	0.082	9	*ICAM1, IL6, TNF, IL1RL1, IL1RL2, CXCL3, CXCL2, NLRP3, CCRL2, CCL20, F3, RASGRP1, IL1B, WFDC1, THBS1, IL1A*	*VCAM1, CCL13, UCN, NUPR1, EPHX2, BDKRB2, PLA2G2D, SELE, ADCYAP1*
**positive regulation of cytokine production**	0.000	13	0.291	5	*IL6, TNF, IL1RL1, IL1RL2, NR4A3, NLRP3, CCL20, RASGRP1, IL1B, THBS1, NLRP10, IL1A, SCAMP5*	*CSF2, UCN, EPX, IFNG, ADCYAP1*
**single organismal cell-cell adhesion**	0.005	14	0.141	9	*ICAM1, IL6, TNF, IL1RL2, NR4A3, NLRP3, MYL9, PKP1, IFNB1, NRARP, TNR, RASGRP1, IL1B, ITGA2B*	*VCAM1, ANXA9, BVES, TENM2, IFNG, LRP6, MPL, PLA2G2D, SELE*

**chemotaxis**	0.002	13	0.551	5	*CCRL2, IL6, CCL20, F3, TNR, CXCL3, CXCL2, SPTBN2, IL1B, NR4A3, THBS1, PTGDR2, FOSL1*	*VCAM1, CCL13, ROBO1, TENM2, IFNG*
**leukocyte migration**	0.001	11	0.275	5	*ICAM1, IL6, TNF, CCL20, CXCL3, CXCL2, IL1B, THBS1, NLRP10, IL1A, ITGA2B*	*VCAM1, CCL13, EPX, IFNG, SELE*
**positive regulation of cellular component movement**	0.002	11	0.801	3	*ICAM1, IL6, TNF, CCL20, F3, CXCL3, CXCL2, THBS1, MYLK, IL1A, ITGA2B*	*WNT5B, IFNG, DDR2*
**positive regulation of cell migration**	0.001	11	0.772	3	*ICAM1, IL6, TNF, CCL20, F3, CXCL3, CXCL2, THBS1, MYLK, IL1A, ITGA2B*	*WNT5B, IFNG, DDR2*
**positive regulation of cell motility**	0.002	11	0.789	3	*ICAM1, IL6, TNF, CCL20, F3, CXCL3, CXCL2, THBS1, MYLK, IL1A, ITGA2B*	*WNT5B, IFNG, DDR2*
**regulation of cell-cell adhesion**	0.004	10	0.761	3	*IL6, TNF, NRARP, IFNB1, TNR, IL1RL2, RASGRP1, IL1B, NR4A3, NLRP3*	*VCAM1, IFNG, PLA2G2D*
**ion transport**	0.500	13	0.003	21	*C15ORF48, CPT1B, ICAM1, SPINK1, KCNJ3, PKD2L2, JSRP1, GRIN2C, IL1B, THBS1, SLCO5A1, SIK1, MYLK*	*C15ORF48, KCNMA1, ARC, SLC38A3, UCN, HNF1A, GNAO1, CACHD1, SLC6A12, BDKRB2, ASIC1, PKD2L1, ADCYAP1, SLC1A2, KCNQ3, CYP27B1, SLC7A3, JSRP1, MCOLN2, PLA2G2D, AKR1C1*
**nitrogen compound transport**	0.692	6	0.001	15	*ABCB9, IL6, TNF, IL1B, BIRC5, UPK3A*	*CSF2, SLC38A3, UCN, HNF1A, SLC6A12, NUP62CL, IGF2BP1, UPK3A, HMGA2, AMN, AZIN2, ADCYAP1, SLC1A2, SLC7A3, IFNG*
**response to lipid**	0.000	18	0.001	16	*E2F1, ICAM1, AR, IL6, TNF, CXCL3, CXCL2, EZH2, NR4A3, NLRP3, NOCT, CCL20, TRIM68, IFNB1, IL1B, WFDC1, THBS1, FOSL1*	*CSF3, CSF2, FGFR4, UCN, WNT5B, RBP1, HMGA2, ALDH3A1, ADCYAP1, VCAM1, SSTR2, CYP27B1, HEY1, IFNG, LRP6, SELE*
